# Maintaining the native gut microbiota of bharal (*Pseudois nayaur*) is crucial in *ex situ* conservation

**DOI:** 10.3389/fmicb.2024.1357415

**Published:** 2024-03-12

**Authors:** Hongmei Gao, Xiangwen Chi, Pengfei Song, Haifeng Gu, Bo Xu, Zhenyuan Cai, Feng Jiang, Bin Li, Tongzuo Zhang

**Affiliations:** ^1^Key Laboratory of Adaptation and Evolution of Plateau Biota, Northwest Institute of Plateau Biology, Chinese Academy of Sciences, Xining, Qinghai, China; ^2^Qinghai Provincial Key Laboratory of Animal Ecological Genomics, Xining, Qinghai, China; ^3^Students’ Affairs Division, Qinghai University, Xining, China; ^4^College of Life Sciences, University of Chinese Academy of Sciences, Beijing, China

**Keywords:** adaption, bharal (*Pseudois nayaur*), *ex situ* conservation, gut microbiota, Qinghai–Tibet Plateau

## Abstract

As wildlife protection continue to strengthen, research on the gut microbiota of wildlife is increasing. Carrying out conservation and research on endangered species in the Qinghai Tibet Plateau plays an important role in global biodiversity conservation. This study utilized 16S rRNA sequencing of fecal samples to investigate the composition, function, and changes of the gut microbiota of bharal in different environments, seasons, and genders. The results showed that Firmicutes and Bacteroidota were the dominant phyla and *UCG-005*, *Bacteroides*, *UCG-010* were the dominant genera of bharal. In the wild, the abundance of Firmicutes increased which was conducive to the decomposition and utilization of cellulose, hemicellulose, and carbohydrate. Due to the variety of food types and nutrition in different seasons, the composition and function of gut microbiota were obviously different between genders. Compared with zoo, higher alpha diversity, a more complex gut microbiota network structure, and stronger metabolic function were conducive bharal to adapting to the wild environment. In the zoo, captive bharals were fed foods rich in high fat and protein, which increased the abundance of Bacteroidota and reduced the alpha diversity of gut microbiota. A fixed diet unified the gut microbiota between genders of bharal. It is very important to pay attention to the impact of captive environments and maintain the native gut microbiota of wildlife.

## 1 Introduction

The Qinghai–Tibet Plateau holds a significant place in the terrestrial ecosystem of the planet due to its distinctive geography. This physical geography unit has fostered a wealth of wildlife resources, making it a crucial area for biodiversity conservation and research, not only in China but also the world (Wang et al., 2015). However, with the global climate change, the warming rate of this region has been twice as fast as the global average, leading to a severe biodiversity crisis in this area, even more severe than other regions (Luo et al., 2015). Therefore, it is crucial to carry out conservation and research on endangered species in the Qinghai–Tibet Plateau to address the decreasing biodiversity issues in China and the world.

To better protect endangered wildlife, China government continuously strengthen the construction of both *in situ* conservation and *ex situ* conservation (Xu et al., 2019). Zoos as important places for *ex situ* conservation, it is a typical artificial enclosure environment. People provides nutritious food for wildlife, and frequent disinfection and sterilization, eliminating all pathogenic and harmful bacteria in environment. However, studies have shown that in an environment where “industrialization” environmental factors are increasing rapidly, they can seriously affect the composition and transmission of animal gut microbiota (Sonnenburg and Sonnenburg, 2019). We speculate that wildlife in zoos is often fed industrial foods such as refined feed, which may lead to imbalances in their gut microbiota and have negative impacts on their health. In a healthy state, the microbial community in the animal gastrointestinal tract remains relatively stable and plays a crucial role in regulating nutritional balance, energy conversion, immune response, adaptive evolution, and growth and development (Simpson et al., 2005; Ezenwa and Gerardo, 2012; Hicks et al., 2018). The gut microbiota has strong plasticity, and food (Ren et al., 2017), season (Gomez et al., 2016), genetic factors (Gomez et al., 2015), and habitat (Amato et al., 2013) can affect their diversity and function.

The bharal (*Pseudois nayaur*), belonging to the Cetartiodactyla, Bovidae, Caprinae, and Pseudois, is a Class II key protected wildlife in China. As a typical alpine animal, bharal is widely distributed and abundant species on the Qinghai–Tibet Plateau and its surrounding areas (Schaller, 1977). It also serves as the main prey for rare carnivores such as snow leopard, which is crucial for maintaining the stability of the ecosystem on the Qinghai–Tibet Plateau and protecting species diversity. In this study, we utilized 16S rRNA gene amplification technology to conduct high-throughput sequencing on fecal samples and discuss the composition, diversity, and function differences of gut microbiota in bharal under various factors. Conducting research on the gut microbiota and function of captive endangered wildlife is not only of great significance for the protection of wildlife, but also provides a new theoretical basis for the future health management of wildlife.

## 2 Materials and methods

### 2.1 Sample collection

In January 2018, 52 fresh fecal samples (WW) of wild bharal were collected from Huashixia Township, Maduo County, Qinghai Province, China. In August of the same year, we collected 45 bharal fecal samples (SW) from the same area. At the same time, we collected 22 fecal samples (WC and SC) of captive bharal at the Qinghai–Tibet Plateau Wildlife Park (longitude: 101.732657, latitude: 36.627469). The freshness of the samples was assessed based on the color and degree of air drying of the feces to ensure high-quality samples. We extracted DNA from the surface of bharal fecal samples using DNeasy Blood & Tissue Kit (QIAGEN), and used sex identification primers (SE47: CAGCCAACCTCCCTCTGC, SE48: CCCGCTTGGCTTGTCTGTTGC) (Weikard et al., 2006) for PCR amplification. The PCR amplification reaction conditions were pre denatured at 94°C for 5 min, Denaturation at 95°C for 30 s, annealing at 60°C for 1 min, and extension at 72°C for 10 min, 30 cycles. Two stripes for male and one stripe for female. We identified the gender of the samples and selected 66 of them for subsequent experiments, including WWM (WW group 17 males), WWF (WW group 13 females), SWM (SW group 4 males), SWF (SW group 10 females), WCM (WC group 8 males), WCF (WC group 3 females), SCM (SC group 8 males), and SCF (SC group 3 females).

### 2.2 Sequencing and annotation

Genomic DNA was extracted from 66 fecal samples using the CTAB method (Yan et al., 2008). The purity and concentration of DNA were tested, and the samples were diluted to 1 ng μl^−1^ with sterile water. Complete PCR amplification (515F and 806R), product mixing and purification. The NEBNext^®^ Ultra™ The IIDNA Library Prep Kit was used for library construction, and the constructed library was quantified using Qubit and Q-PCR. After library qualification, the Ion Torrent S5 XL was used for machine sequencing. Split each sample data from the offline data based on the barcode sequence and PCR amplification primer sequence. The barcode and primer sequence were removed, and used FLASH (V1.2.11) (Magoč and Salzberg, 2011) software concatenated the reads of the sample to obtain Raw Tags. Subsequently, Fastp software was used to perform quality control on the raw tags obtained, resulting in high-quality Clean Tags. Finally, we used Usearch software to compare clean tags with the database to detect and remove chimeras (Haas et al., 2011), and effective Tags were obtained. At same time, we used the DADA2 module in QIIME2 software to reduce the noise and filtered out sequences with an abundance less than 5 to obtain the Amplicon Sequence Variables (ASVs) information table. Next, we used the classify sklearn module to compare the obtained ASVs with the Silva138.1 database and obtain species information for each ASV. QIIME2’s classify sklearn algorithm (Bokulich et al., 2018) was used to annotate each ASV with a pre trained Naive Bayes classifier, and obtained the corresponding species information and the distribution of species based on abundance.

At the same time, alpha diversity, and the abundance of ASVs were also calculated to determine species richness and evenness within each sample. Phylogenetic trees were constructed by aligning multiple sequences of ASVs, and community structure differences between groups were explored using PCoA (Principal Co-ordinates Analysis) and NMDS (Non-metric Multidimensional Scaling). Tax4Fun functional prediction was achieved using the nearest neighbor method based on minimum 16S rRNA sequence similarity. Specifically, the entire 16S rRNA gene sequence was extracted from prokaryotic organisms in the KEGG database and compared to the SILVA SSU Ref NR database using the BLASTN algorithm (BLAST bitscore >1,500) to establish a correlation matrix. The KEGG database was annotated using the UProC and PAUDA methods, and the entire genome functional information of prokaryotes was assigned to the SILVA database, enabling functional annotation of the SILVA database.

### 2.3 Data analysis

Alpha diversity can reflect the diversity of gut microbiota composition, and we choose Observed_Otus index (the number of observed specifications) to reflect the microbial community richness, Shannon index to reflect the microbial community diversity, and Pielou_e index (Pielou’s evenness index) to reflect the species evenness of microbial community. We used Qiime2 to calculate these alpha diversity indexes and plot rarefaction curves. Wilcoxon rank sum test was used to compare the alpha diversity indices among different groups to determine significant differences. To compare β diversity among groups, we used two similarity distance algorithms, Unweighted Unifrac, and Weighted Unifrac to calculate the distance between each sample at the ASVs level. An Anosim (analysis of similarities) and Adonis (permutational manova) were used to perform inter group (SCF-SCM, SWF-SWM, WCF-WCM, and WWF-WWM) difference tests (R Software, Version 3.5.3) (packages “vegan”). All species composition and functional abundance data were tested for normality using the Shapiro–Wilk test function in the *R*. Results with a *value of p* greater than 0.05 indicate that our data distribution follows normality. *T*-tests were used to analyze differences in the gut microbiota of bharal at the phylum and genus levels, based on factors such as environment, gender, and season. Due to unequal variances when comparing two groups, bilateral tests were chosen. We performed co-occurrence network analysis at the genus level and excluded genera with relative abundance less than 0.01 to reduce the impact of rare bacterial genera on the results. After calculating the network diagram in R (packages “igraph,”), we used Gephi (V0.10.1) software to adjust the image and calculated parameters such as average degree, average weighted degree, network diameter, average path length, density, and so on. Based on the results of modularity, we determined the size and color of each node in the network diagram. Other plots shown in this study were drawn using R (Version 3.5.3) and various software packages including “ggplot2,” “reshape2” (Stacked histograms), “VennDiagram” (Petal diagram), “ggpubr” (box plot), and “vegan,” “ggplot2” and “ade4” (PCoA and NMDS).

## 3 Results

### 3.1 Overview of gut microbiota in bharal

**Figure 1:**
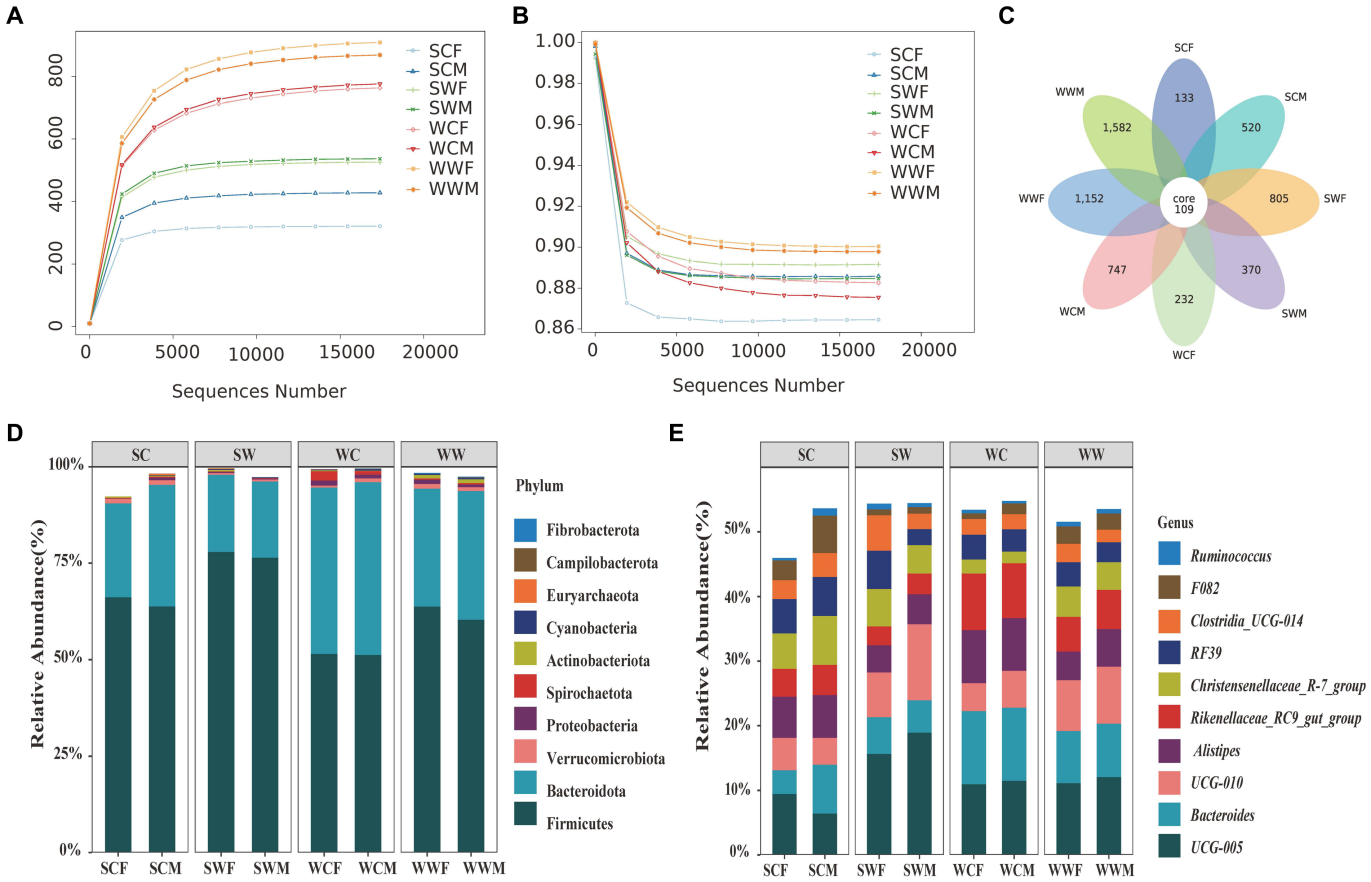
Rarefaction curves of community richness **(A)** and community evenness **(B)** of gut microbiome in bharal. Petal diagram of the number of ASVs among different groups **(C)**. Stacked histograms of the relative abundance of gut microbiota at phylum level **(D)** and genus level **(E)** of bharal.

After sequencing 66 bharal fecal samples and filtering out low-quality base sequences, we obtained a total of 5,577,112 raw data for preliminary quality control. After filtering out chimeras, 5,236,180 high-quality sequences (clean reads) were used for subsequent analysis, with an average read length of 370 bp. The percentage of clean reads to raw reads was 93.92%. We randomly selected sequencing data from bharal fecal samples as the horizontal axis, and used microbial richness and community evenness index (at the ASVs level) as the vertical axis to draw rarefaction curve for bharal sequencing samples. As the sequencing depth continues to increase, the actual observed values of ASVs continue to rise (Figure 1A) and community evenness continue to decline (Figure 1B). When the sequencing quantity was 15,000, the curve gradually flattens out, and indicating that the quantity and quality of this sequencing were reliable, and the sequencing depth was sufficient to obtain most of the microbial information in the bharal fecal samples. The sequencing results can well reflect the diversity of gut microbiota in different environments, seasons, and genders of bharal.

In total 5,650 effective ASVs were identified in all samples, among them, the number of ASVs in the WWF (896.27 ± 112.91, mean ± SD) was the highest, followed by WWM (874.4 ± 98.26), WCM (775.88 ± 89.17), WCF (763 ± 49.07), SWM (536.5 ± 36.88), SWF (525.6 ± 55.64), SCM (427.5 ± 111.38), and SCF (320.67 ± 10.37), respectively. Among these groups, only 109 were shared, representing a mere 1.93% of the total ASVs. The highest proportion of ASVs were to be unique to WWM, with 1,582, accounting for 28% of the total ASVs. Subsequently, WWF (1,152, 20.39%), SWF (805, 14.25%), WCM (747, 13.22%), SCM (520, 9.2%), SWM (370, 6.55%), WCF (232, 4.11%), and SCF (133, 2.35%) were also identified (Figure 1C).

### 3.2 Composition and abundance analysis of gut microbiota in bharal

At the phylum level, the top 10 phyla of bacteria accounted for 98.17% of the gut microbiota. Firmicutes made up the highest proportion of the total bacterial content at 63.85% ± 10.87% (mean ± SD), followed by Bacteroidota (31.17% ± 9.62%), Verrucomicrobiota (0.90% ± 0.95%), Proteobacteria (0.78% ± 1.31%), Actinobacteriota (0.56% ± 0.75%), Spirochaeta (0.44% ± 0.61%), Cyanobacteria (0.21% ± 0.20%) Euryarchaeota (0.10% ± 0.17%), Campilobacterota (0.09% ± 0.19%), and Fibrobacterota (0.08% ± 0.20%). At the genus level, the top 10 genera were *UCG-005* (11.92% ± 3.72%), *Bacteroides* (7.82% ± 3.25%), *UCG-010* (7.23% ± 4.29%), *Alistipes* (5.72% ± 2.34%), *Rikenellaceae_RC9_gut_group* (5.45% ± 2.46%), *Christensenellaceae_R-7_group* (4.69% ± 2.32%), *RF39* (4.14% ± 2.10%), *Clostridia_UCG-014* (2.98% ± 1.91%), *F082* (2.47% ± 1.88%), and *Ruminococcus* (0.72% ± 0.63%). To visually represent the distribution of the top 10 bacterial in each sample, we created stacked histograms showing the relative abundance of gut microbiota at phylum level and genus level for each group (Figures 1D,E).

**Figure 2:**
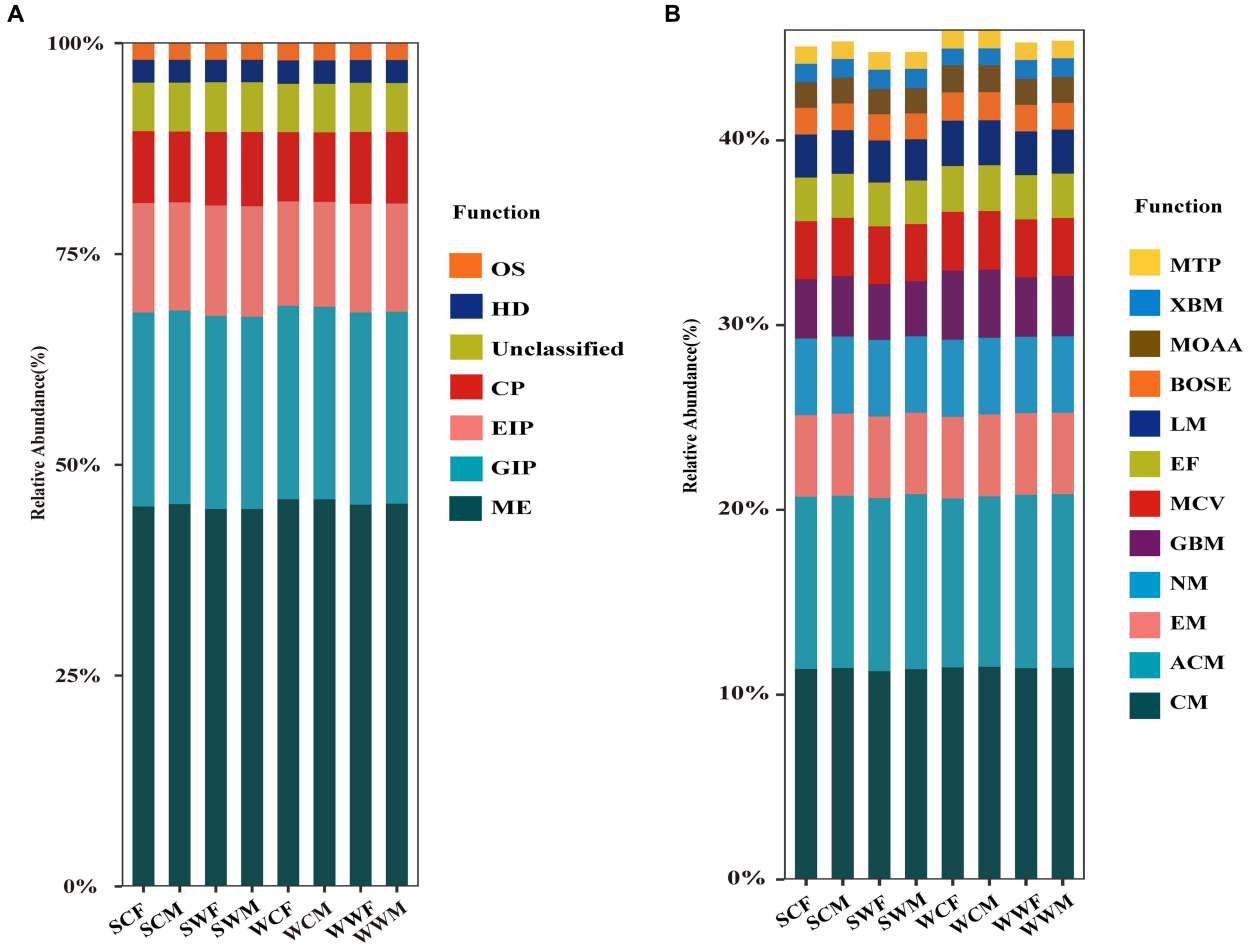
Relative abundance histogram of gut microbiota at level 1 **(A)** and level 2 **(B)** based on the KEGG database.

According to the functional predictions, Metabolism (ME) was the most important function at level 1 (Figure 2A), accounting for 45.30% ± 0.47% of the total functions, followed by Genetic Information Processing (GIP) (22.86% ± 0.15%), Environmental Information Processing (EIP) (12.85% ± 0.29%), Cellular Processes (CP) (8.47% ± 0.22%), Human Diseases (HD) (2.71% ± 0.04%), and Organismal Systems (OS) (1.97% ± 0.04%). At level 2 (Figure 2B), we conducted a comparative analysis of the main metabolic functions, including Carbohydrate Metabolism (CM) (11.41% ± 0.11%), Amino Acid Metabolism (ACM) (9.36% ± 0.12%), Energy Metabolism (EM) (4.43% ± 0.03%), Nucleotide Metabolism (NM) (4.13% ± 0.03%), Glycan Biosynthesis and Metabolism (GBM) (3.28% ± 0.27%), Metabolism of Cofactors and Vitamins (MCV) (3.14% ± 0.04%), Enzyme Families (EF) (2.41% ± 0.04%), Lipid Metabolism (LM) (2.34% ± 0.08%), Biosynthesis of Other Secondary Metabolites (BOSE) (1.46% ± 0.05%), Metabolism of Other Amino Acids (MOAA) (1.41% ± 0.03%), Xenobiotics Biodegradation and Metabolism (XBM) (0.99% ± 0.06%) and Metabolism of Terpenoids and Polyketides (MTP) (0.94% ± 0.04%).

### 3.3 Analysis of differences between different groups

**Figure 3:**
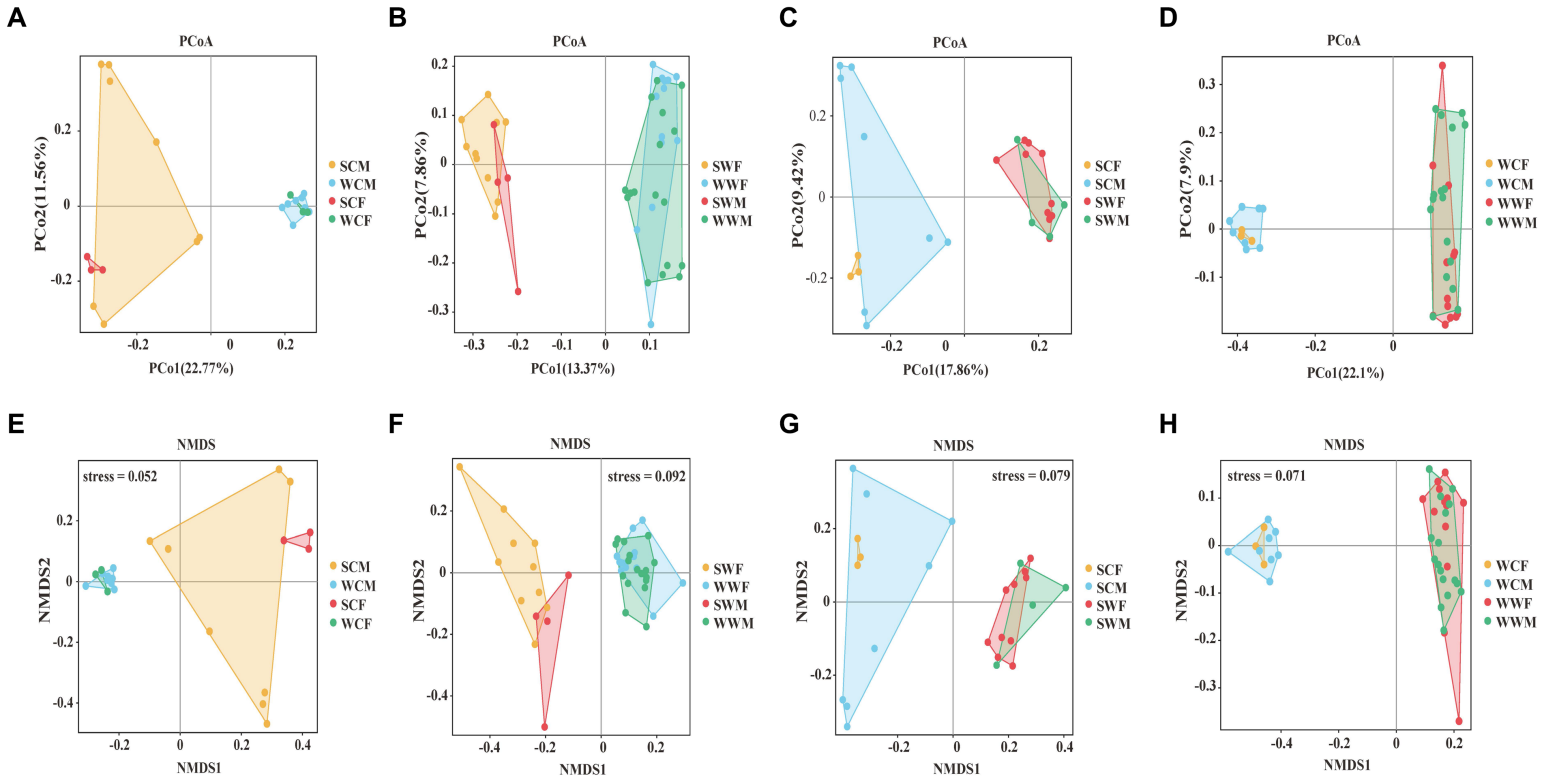
PCoA analysis **(A, B, C, D)** and NMDS analysis **(E, F, G, H)** of gut microbiome in different groups.

In the PCoA analysis, for the captive environment, samples from each group clustered, summer and winter groups were clearly separated, but the separation of samples between different gender groups were no significant difference during the same season (Figure 3A). In the wild environment, the summer and winter samples were clearly separated (Figure 3B), but within the same season, the samples of different gender groups were more distinctly separated in summer (Figure 3C), whereas they were mixed in winter (Figure 3D). In the NMDS analysis, the stress values are 0.052, 0.092, 0.079, and 0.071 (Figures 3E–H), respectively, which accurately reflected the degree of difference between samples. These prove that our grouping was reasonable and the difference results were reliable. The Anosim and Adonis analyses based on the Weighted Unifrac and Unweighted Unifrac distance algorithms jointly revealed that there were no significant differences observed among gender groups. However, it was observed that significant differences (adjust *p* < 0.05) exist among environmental groups. Among seasonal factors, except for SCF-WCF, all other groups showed significant differences (adjust *p* < 0.05) (Supplementary Appendix A). Based on the above analysis, we discovered significant differences in the gut microbiota of bharal across different seasons and environments. Therefore, in subsequent analyses, we will examine different factors.

### 3.4 The impact of gender on gut microbiota in bharal is small

**Figure 4:**
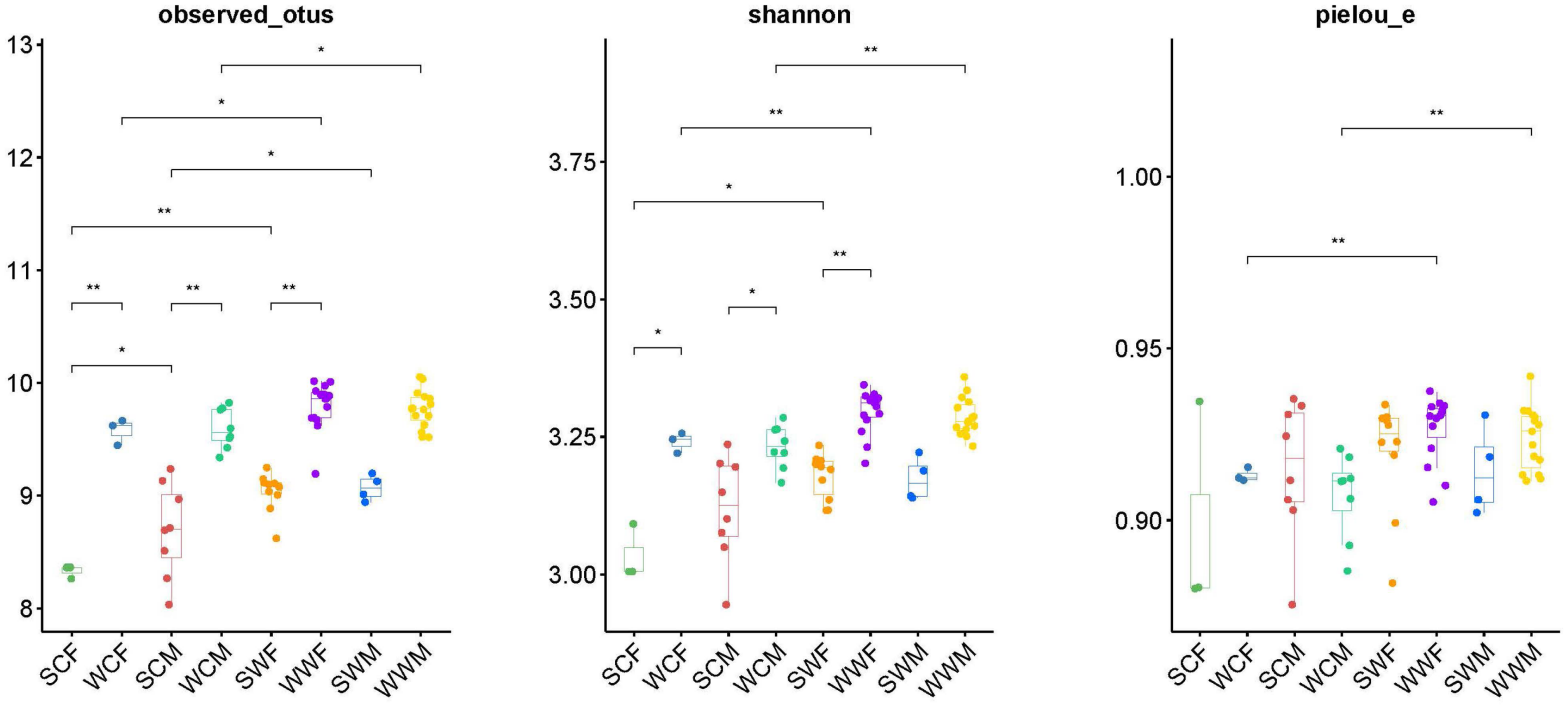
Differences analysis of alpha diversity (Observed_Otus index, Shannon index, and Pielou_e index). *p < 0.05, **p < 0.01.

In the alpha diversity analysis of bharal gut microbiota (Figure 4), there was no significant difference in the Pielou_e index and Shannon index, but the Observed_otus index was higher in SCM than in SCF. When analyzing inter-group differences between dominant phyla and genera, we found no significant difference in dominant phyla between groups. However, at the genus level, *UCG-005* and *UCG-002* in SCF were higher than those in SCM (*p* < 0.05), while *Bacteroides*, *F082*, and *Family_XIII_AD3011_group* in SCM were higher than in SCF (*p* < 0.05). In SWF, *RF39* and *Clostridia_UCG-014* were higher than in SWM (*p* < 0.05), while *UCG-014* was higher in SWM than in SWF (*p* < 0.05). In WWM, *Alistipes* was significantly higher than in WWF (*p* < 0.05), while *Clostridia*_*UCG-014*, *Monolobus*, and *UCG-002* were higher in WWF than in WWM (*p* < 0.05) (Figure 3). In functional analysis, at level 1, we found that only HD showed a higher WWM than WWF (*p* < 0.05), with no significant differences in other functions. At level 2, AAM shows that SWM was higher than SWF, and MCV, MTP and NM showed that SWF was higher than SWM (*p* < 0.05), and there was no significant difference in other functions (Supplementary Appendices B,C).

### 3.5 There are significant seasonal changes in gut microbiota of bharal

In the analysis of alpha diversity (Figure 4), whether in captivity or the wild, we observed a higher observed_otus index and Shannon index in winter compared to summer (*p* < 0.05). This suggests that the abundance and diversity of the gut microbiota of bharal was significantly higher in winter than in summer. In terms of inter-group differences, at the phylum level, Bacteroidota was higher in winter (*p* < 0.05), while Firmicutes was higher in summer (*p* < 0.05). At the genus level, *F082* showed higher abundance in SC group (*p* < 0.05), but higher in WW group (*p* < 0.05). *Rikenellaceae_RC9_gut_group* was higher in WC group than in SC group (*p* < 0.05), *UCG-005* was higher in SW group than in WW group (*p* < 0.05), *Bacteroides* and *Rikenellaceae_RC9_gut_group* were higher in WW group than in SW group (*p* < 0.05).

Through functional prediction, it was observed that at level 1, ME, HD, and OS were higher in winter than in summer (*p* < 0.05), while CP was higher in summer than in winter (*p* < 0.05). GIP was only higher in SWF than WWF (*p* < 0.05), while EIP was higher in SWM than WWM (*p* < 0.05). At level 2, in the captive group, EM, EF and MOAA were higher in winter than in summer (*p* < 0.05). In the wild, BOSM, CM, EF, GBM, LM, and MOAA were higher in winter than in summer (*p* < 0.05). XBM was higher in summer than in winter (*p* < 0.05) in both in wild and captive groups (Supplementary Appendices B,C).

### 3.6 The environment has a significant impact on the gut microbiota of bharal

In the alpha diversity analysis (Figure 4), we observed that the observed_otus, Shannon index, and Pielou_e evenness index were significantly higher in the wild group compared to the captive group for both summer and winter (*p* < 0.01). When analyzing inter-group differences in dominant phyla and genera, all groups showed significantly higher levels of Bacteroidota in the captive compared to the wild, and significantly higher levels of Firmicutes in the wild compared to the captive, except for SCF and SWF groups. In terms of common bacterial genera, *F082* showed higher levels in the SC (*p* < 0.05), but higher levels in the WW (*p* < 0.05). *UCG-002* and *Rikenellaceae_RC9_gut_group* showed higher abundance in the captive environment compared to the wild in both seasons (*p* < 0.05). In winter, *Christenselelaceae_R-7_group*, *Monoglobus*, and *NK4A214_group* showed higher performance in the wild compared to the captive (*p* < 0.05).

Through functional prediction, we found that at level 1, ME, HD, and OS were higher in captivity than in the wild. CP was higher in wild than in captive. EIP is higher in WW than in WC, and OS was higher in WC than in WW. At level 2, BOSM, CM, EM, EF, GBM, LM, MCV, MOAA, and MTP were significantly higher in captive than in wild. However, when comparing the WCF-WWF group, BOSM, EF, GBM, MOAC, and MTP were higher in WWF than in WCF, and XBM was higher in the wild than in the captive in both seasons (Supplementary Appendices B,C).

### 3.7 Analysis of co-occurrence network of gut microbiota in bharal

**Figure 5:**
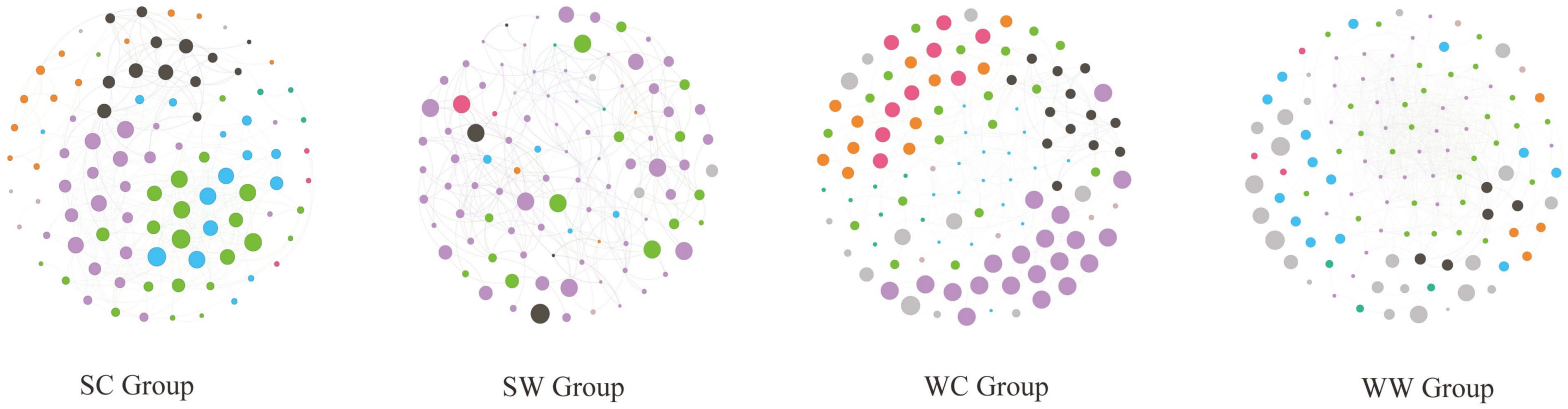
The structure of gut microbial networks in bharal among different groups.

The analysis revealed that the structure of gut microbial networks in bharal varied across seasons and environments, exhibiting varying levels of complexity. Specifically, the WW group had the most complex network structure, with 525 edges, followed by the SC group (337 edges), WC group (297 edges), and SW group (245 edges). Interestingly, the SC and WC groups had no negative edges, while SW had only 2 and WW had 190. The network diagram also showed that the modules of the network structure were distinct for each group. For instance, in the WC group, the purple module (20 nodes) was the most significant, followed by the green (19 nodes) and blue (18 nodes) modules. However, only *UCG-010* (15) and *UCG-009* (11) from the blue module had more than 10 edges, indicating they were part of the core area of the entire network. In the SC group, the purple (23 nodes), green (20 nodes), and blue (15 nodes) modules were the most important, followed by the black module (13 nodes). The genera with lines greater than or equal to 10 are *Christensenellaceae_R-6_group* (17 edges, blue module), *Family_XIII_AD3010_group* (15 edges, green module), *F081*(13 edges, green module), *UCG-001* (13 edges, green module), *NK4A213_group* (10 edges, purple module), *Methanobrevibacter* (10 edges, blue module), and *Candidatus_Soleaferrea* (10 edges, green module). In the SW group, the proportion of purple modules was relatively larger (68 nodes), followed by green modules (14 nodes). However, when looking at the statistics for the number of edges, only *Christensenellaceae_R-7_group* (10 nodes) in the purple module was found with 10 edges, while the remaining bacteria had less than 10 edges. In the WW group, compared to other groups, there were the most edges and the most complex structure. The modules with the most nodes were green (30 nodes) and purple (30 nodes). In the edge count statistics, the genera with purple modules with more than 10 edges were *Christensenellaceae_R-7_group* (35 edges), *RF39* (30 edges), *NK4A214_group* (30 edges), *Clostridia_UCG-014* (28 edges), *UCG-002* (28 edges), *Monoglobus* (21 edges), *UCG-009* (14 edges), *Bacteroides* (13 edges), and *Lachnospiraceae_AC2044_group* (10 edges). For green module, the following genera had more than 10 edges were *Clostridia_vadinBB60_group* (19 edges), *UCG-010* (19 edges), *Victivallaceae* (18 edges), *Candidatus_Soleaferrea* (17 edges), *Izemoplasmatales* (17 edges), *Eubacterium_coprostanoligenes_group* (16 edges), *Phascolarctobacterium* (16 edges), *Ruminococcus* (13 edges), and *Alistipes* (10 edges) (Figure 5).

## 4 Discussion

Due to the small population and difficult-to-obtain the samples of endangered animals, collecting fresh feces through non-contact sampling has gradually become the best research method for conservation biology (Wei et al., 2019; Guo et al., 2020; Zhang et al., 2022). At the phylum level, the dominant phyla (abundance >1%) of bharal in different genders, seasons, and environments are Firmicutes and Bacteroidota, which is consistent with the results of most herbivorous gut microbiota (Chi et al., 2019; Li et al., 2022; Gao et al., 2023). As the dominant microbiome of bharal, these bacterial helps host to digest and decompose cellulose and hemicellulose in diet (Wu et al., 2016), which is great significance to the digestion, decomposition, and metabolism of bharal. In the alpha diversity analysis, we observed that there were significant differences based on environmental and seasonal factors. The diversity of gut microbiota in winter was higher than that in summer, while in the wild was higher than that in the captive, which is consistent with previous researches (Chi et al., 2019; Gao et al., 2020; Jiang et al., 2022). Alpha diversity is a quantitative indicator that indicates the diversity, stability, and community composition structure of the host’s gut microbiota. It is also regarded as a crucial indicator of the host’s overall health status (Shanahan, 2010; Lang et al., 2018). We believe that compared with captive bharal, the gut microbiota of wild bharal is more stable, which enhances wild populations’ survival and adaptability.

In the analysis of gender impact, we found that even though the genera with differences between female and male individuals are different, they generally exhibit a pattern. The genera with higher relative abundance in the gut microbiota of female bharals (*UCG-005*, *UCG-002*, *RF39*, *and Clostridia_UCG-014*) belong to Firmicutes, while the genera with higher relative abundance in male bharals (*Bacteroides*, *F082*, *Alistipes*, and so on) belong to Bacteroidota. Previous studies have also shown similar results (Wang et al., 2020). We attribute these differences to variations in the physiological structure and sex steroid hormones such as estradiol and testosterone (Flores et al., 2012; Kaliannan et al., 2018). In captive environment, the gut microbiota function remained the same. However, in the wild, during winter when food is scarce, male, and female bharal exhibit a high degree of overlap in their dietary, resulting there was no difference in the structure and function of gut microbiota. However, during summer, there is a significant difference in food composition between male and female (Zhang, 2013). In the case, the composition and function of the gut microbiota of male and female bharal have changed. Male bharals prefer to consume high fiber foods, and Bacteroidota is beneficial to degrade cellulose (Li et al., 2021). Female bharals tend to consume high-quality food resources, Firmicutes increase the degradation of carbohydrates, fats, and proteins (Wu et al., 2020). These changes not only play an important role in the digestion and decomposition of different types of food, but also in adjusting functions that better adapt to the wild environment based on the host’s characteristics.

In the analysis of seasonal impact, we found that Bacteroidota was higher in winter, while Firmicutes was higher in summer. This could be due to the higher levels of crude protein and fat in summer (Fan, 2002). The increase in Firmicutes abundance can benefit bharal to degrade carbohydrates (especially polysaccharides) and other substances such as protein, improve nutrient utilization, and maintain a balanced gut microbiota (Liu et al., 2020). At the genus level, *UCG-005* and *RF39* increase in abundance during summer, which were related to cellulose degradation and promotes the production of butyrate (Liang et al., 2021). The abundance of *Rikenellaceae_RC9_gut_group* and *Bacteroides* were higher in winter. Previous studies have shown that *Rikenellaceae_RC9_gut_group* was involved in the synthesis of dimethyl acetals during rumen fermentation, and was associated with the production of short chain fatty acids, especially propionate, which may enhance ruminant productivity in winter (Conte et al., 2022). The increased of *Bacteroides* was beneficial for improving host metabolism, regulating bile acids, short chain fatty acids, sugars, proteins, fat metabolism, and reducing fat deposition (Wahlström et al., 2016). Through these microbiota changes, the utilization and adaptability of winter bharal to food resources were enhanced (Tremaroli and Backhed, 2012). In functional analysis, at level 1 and level 2, most metabolic functions were higher in winter. Compared to the captive group, the wild groups had more functions that increase in abundance during winter. The enhancement of metabolism and other functions is great significance for bharal to adapt the wild environment. It is worth noting that XBM was higher in summer than in winter. We speculate that there are abundant types of secondary compounds in summer food, and the enhancement of this function is conducive to the full utilization of food resources.

When analyzing differences in environmental factors, we found that captive bharals were fed artificial feed with high fat and protein content (Gao et al., 2020). Previous studies have shown that high crude fat content in the diet can lead to a decrease in the number of Bacteroidetes in the host and an increase in the number of Firmicutes (Ni, 2018), which is consistent with our findings. At the genus level, *F082* appeared to be more abundant in the captive group than wild group during summer, and may play a key role in the digestion of non-structural carbohydrates (Yi et al., 2022). *UCG-002* and *Rikenellaceae_RC9_gut_group* were increased which is conducive to the degradation and utilization of food and promotes the production of compounds such as acetate and propionate (Shabat et al., 2016; Liang et al., 2021). *Christensenellaceae_R-7_group*, *Monoglobus*, and *NK4A214_group* showed higher abundance in the wild group than in the captive group, which are mainly involved in the degradation of cellulose and hemicellulose and have a positive correlation with propionate, butyrate, and isobutyrate (Dai et al., 2022). These bacteria can promote acetate fermentation in the rumen, which usually has a slow energy supply but can sustain bharal in wild winter environments for a long time (Wu et al., 2022). In functional analysis, at level 1, the ME in the captive group was stronger than that in the wild group. At level 2, the enhanced metabolic functions in captive bharal were mainly related to material metabolism, likely due to the abundance of captive food. Enhancements in metabolic functions related to lipids, amino acids, and proteins can help captive bharal better utilize these foods. However, for wild bharal, during the winter when food is scarce, their enhanced metabolic functions tend to focus on the biosynthesis of metabolites, using limited food resources to synthesize useful compounds for themselves.

In co-occurrence network analysis, we removed rare bacteria (abundance <0.01%) to minimize their impact on the network structure (Galand et al., 2009). During winter, wild bharal had the most edges, the shortest average path length, and the most complex network structure. They also formed the most modules. We believe that the primary reason for these results is the lack of food resources in the wild during winter, which increases the interaction between microbial communities to optimize resource utilization and cope with harsh environments. In contrast, the captive bharal in summer should be the most comfortable compared to other groups, with a simpler relationship and structure between microbial communities. However, this simpler structure may also make it easier for some bacteria to be damaged, and their intestinal environment is more susceptible to damage than other groups. Through modular analysis and edge counting, we identified some key genera in the gut microbiota co-occurrence network of wild and captive bharal under different environments and seasons, which play a vital role in maintaining each functional module. Changes in the gut microbiota network structure of bharal are also a result of adaptation to environmental and seasonal changes.

Our findings indicate that the environment and season affect the gut microbiota of bharal. The captive environment has a significant impact on the gut microbiota composition and function of bharal. We need to attach the importance of maintaining the native gut microbiota of wild animals in captive environment. However, relying solely on 16S sequencing data to predict the gut microbiota function of bharal is inadequate. Therefore, we plan to use metagenomic sequencing to further analyze the gut microbiota function and metabolic pathways of bharal in different genders, seasons, and environments, providing a more comprehensive understanding of the impact of different factors on the gut microbiota of bharal.

## Data availability statement

The datasets presented in this study can be found in online repositories. The data presented in the study are deposited in the NCBI, accession number PRJNA1006445.

## Ethics statement

The animal studies were approved by the Northwest Institute of Plateau Biology, Chinese Academy of Sciences animal ethics committee. The studies were conducted in accordance with the local legislation and institutional requirements. Written informed consent was obtained from the owners for the participation of their animals in this study.

## Author contributions

HGa: Conceptualization, Data curation, Formal analysis, Funding acquisition, Investigation, Methodology, Project administration, Resources, Software, Supervision, Validation, Visualization, Writing – original draft, Writing – review & editing. XC: Conceptualization, Data curation, Formal analysis, Investigation, Methodology, Writing – review & editing. PS: Conceptualization, Data curation, Formal analysis, Investigation, Methodology, Writing – review & editing. HGu: Conceptualization, Data curation, Investigation, Project administration, Writing – review & editing. BX: Conceptualization, Data curation, Investigation, Methodology, Writing – review & editing. ZC: Data curation, Methodology, Project administration, Validation, Writing – review & editing. FJ: Conceptualization, Data curation, Project administration, Writing – review & editing. BL: Data curation, Investigation, Methodology, Writing – review & editing. TZ: Conceptualization, Data curation, Formal analysis, Funding acquisition, Methodology, Project administration, Resources, Writing – review & editing.
