# Supplementary material for: Maintaining the native gut microbiota of bharal (Pseudois nayaur) is crucial in ex situ conservation
Source: Front Microbiol. 2024 Mar 12;15:1357415. doi: 10.3389/fmicb.2024.1357415 (PMC10963425; doi:10.3389/fmicb.2024.1357415)
Supplement: Supplementary file 1 [file Table_1.DOCX]

**Appendix A The ANOSIM and Adonis analyses based on the Weighted Unifrac and Unweighted Unifrac distance algorithms jointly**

| **Factor** | **Groups** | **Weighted unifrac** | | | | **Unweighted unifrac** | | | |
| --- | --- | --- | --- | --- | --- | --- | --- | --- | --- |
|  |  | **ANOSIM** | | **Adonis** | | **ANOSIM** | | **Adonis** | |
|  |  | **R** | **adjust *P*-value** | **R^2^** | **adjust *P*-value** | **R** | **adjust *P*-value** | **R^2^** | **adjust *P*-value** |
| Gender | SCF-SCM | 0.159 | 0.280 | 0.166 | 0.140 | 0.190 | 0.119 | **0.146** | 0.061 |
|  | SWF-SWM | 0.080 | 0.320 | 0.136 | 0.123 | 0.181 | 0.19 | 0.099 | 0.068 |
|  | WCF-WCM | 0.056 | 0.383 | 0.086 | 0.520 | 0.088 | 0.232 | 0.102 | 0.412 |
|  | WWF-WWM | 0.093 | 0.066 | 0.043 | 0.257 | 0.083 | 0.104 | **0.048** | 0.613 |
| Environment | **SCF-SWF** | 0.444 | 0.066 | **0.190** | **0.0210*** | **0.963** | **0.009*** | **0.230** | **0.004**** |
|  | **SCM-SWM** | **0.621** | **0.036*** | **0.367** | **0.006**** | **0.726** | **0.009**** | **0.213** | **0.003**** |
|  | **WCF-WWF** | **0.661** | **0.040*** | **0.295** | **0.006**** | **0.990** | **0.009**** | **0.236** | **0.005**** |
|  | **WCM-WWM** | **0.532** | **0.015*** | **0.314** | **0.040**** | **0.960** | **0.009**** | **0.220** | **0.003**** |
| Season | **SCF-WCF** | 1.000 | 0.132 | 0.642 | 0.133 | 1.000 | 0.148* | 0.450 | 0.109 |
|  | **SCM-WCM** | **0.680** | **0.015*** | **0.409** | **0.004**** | **0.705** | **0.009**** | **0.245** | **0.003**** |
|  | **SWF-WWF** | **0.324** | **0.015*** | **0.247** | **0.004**** | **0.789** | **0.009**** | **0.200** | **0.003**** |
|  | **SWM-WWM** | **0.666** | **0.015*** | **0.265** | **0.006**** | **0.900** | **0.009**** | **0.149** | **0.004**** |

*：*P* < 0.05，**：*P* < 0.01；

**Appendix B** **Different functions of gut microbiota at level 1 based on KEGG database**

| **Factor** | **Groups** | **ME** | **GIP** | **EIP** | **CP** | **HD** | **OS** |
| --- | --- | --- | --- | --- | --- | --- | --- |
| Gender | SCF-SCM | —— | —— | —— | —— | —— | —— |
|  | SWF-SWM | —— | —— | —— | —— | —— | —— |
|  | WCF-WCM | —— | —— | —— | —— | —— | —— |
|  | WWF-WWM | —— | —— | —— | —— | 0.0495^WWM^ | —— |
| Environment | SCF-SWF | 0.0022^SCF^ | —— | —— | 0.0024^SWF^ | 0.0077^SCF^ | —— |
|  | SCM-SWM | 0.0282^SCM^ | —— | —— | 0.0038^SWM^ | 0.0318^SCM^ | —— |
|  | WCF-WWF | 0.0468^WCF^ | —— | 0.0490^WWF^ | 0.0167^WWF^ | 0.0052^WCF^ | 0.0006^WCF^ |
|  | WCM-WWM | 0.0007^WCM^ | —— | 0.0021^WWM^ | 0.0011^WWM^ | 0.0022^WCM^ | 0.0012^WCM^ |
| Season | SCF-WCF | —— | —— | 0.0439^SCF^ | 0.0215^SCF^ | 0.0247^WCF^ | 0.0046^WCF^ |
|  | SCM-WCM | 0.016^WCM^ | —— | 0.0142^SCM^ | —— | 0.0493^WCM^ | 0.0091^WCM^ |
|  | SWF-WWF | 0.0008^WWF^ | 0.0066^SWF^ | —— | 0.0006^SWF^ | 0.0004^WWF^ | 0.0073^WWF^ |
|  | SWM-WWM | 0.0033^WWM^ | —— | 0.0127^SWM^ | 0.0020^SWM^ | 0.0080^WWM^ | 0.0105^WWM^ |

ME: Metabolism; GIP: Genetic Information Processing; EIP: Environmental Information Processing; CP: Cellular Processes; HD: Human_Diseases; OS: Organismal_Systems; The letters represent the group with significantly higher abundance.

**Appendix C Different Metabolism functions of gut microbiota at level 2 based on KEGG database**

| Factor | Groups | ACM | BOSE | CM | EM | EF | GBM | LM | MCV | MOAA | MTP | NM | XBM |
| --- | --- | --- | --- | --- | --- | --- | --- | --- | --- | --- | --- | --- | --- |
| Gender | SCF-SCM | —— | —— | —— | —— | —— | —— | —— | —— | —— | —— | —— | —— |
|  | SWF-SWM | 0.0133^SWM^ | —— | —— | —— | —— | —— | —— | 0.0464^SWF^ | —— | 0.0320^SWF^ | 0.0253^SWF^ | —— |
|  | WCF-WCM | —— | —— | —— | —— | —— | —— | —— | —— | —— | —— | —— | —— |
|  | WWF-WWM | —— | —— | —— | —— | —— | —— | —— | —— | —— | —— | —— | —— |
| Environment | SCF-SWF | —— | 0.0319^SCF^ | 0.0006^SCF^ | —— | —— | 0.0305^SCF^ | 0.0032^SCF^ | —— | 0.0005^SCF^ | —— | —— | 0.0325^SWF^ |
|  | SCM-SWM | 0.0017^SWM^ | 0.0235^SCM^ | —— | —— | 0.0381^SCM^ | 0.0267^SCM^ | 0.0023^SCM^ | 0.0088^SCM^ | 0.0031^SCM^ | 0.0014^SCM^ | —— | —— |
|  | WCF-WWF | 0.0014^WCF^ | 0.0486^WWF^ | —— | —— | 0.0002^WWF^ | 0.0412^WWF^ | —— | —— | 0.0121^WWF^ | 0.0155^WWF^ | —— | 0.0169^WWF^ |
|  | WCM-WWM | 0.0174^WWM^ | 0.0010^WCM^ | 0.0477^WCM^ | 0.0306^WCM^ | 0.0025^WCM^ | 0.0005^WCM^ | 0.0020^WCM^ | 0.0019^WCM^ | 0.0005^WCM^ | 0.0001^WCM^ | —— | 0.0023^WWM^ |
| Season | SCF-WCF | 0.0082^SCF^ | —— | —— | 0.0421^WCF^ | 0.0010^WCF^ | —— | —— | —— | 0.0134^WCF^ | —— | —— | 0.0230^SCF^ |
|  | SCM-WCM | —— | 0.0076^WCM^ | —— | 0.0010^WCM^ | 0.0006^WCM^ | 0.0224^WCM^ | —— | —— | 0.0129^WCM^ | —— | —— | 0.0104^SCM^ |
|  | SWF-WWF | —— | 0.0034^WWF^ | 0.0045^WWF^ | 0.0051^WWF^ | 0.0123^WWF^ | 0.0010^WWF^ | 0.0005^WWF^ | —— | 0.0018^WWF^ | —— | —— | 0.0010^SWF^ |
|  | SWM-WWM | 0.0410^SWM^ | 0.0030^WWM^ | 0.0259^WWM^ | —— | 0.0000^WWM^ | 0.0049^WWM^ | 0.0084^WWM^ | 0.01367^WWM^ | 0.0010^WWM^ | 0.0429^WWM^ | —— | 0.0009^SWM^ |

ACM: Amino_acid_metabolism; BOSE: Biosynthesis_of_other_secondary_metabolites; CM: Carbohydrate_metabolism; EM: Energy_metabolism; EF: Enzyme_families; GBM: Glycan_biosynthesis_and_metabolism; LM: Lipid_metabolism; MCV: Metabolism_of_cofactors_and_vitamins; MOAA: Metabolism_of_other_amino_acids; MTP: Metabolism_of_terpenoids_and_polyketides; NM: Nucleotide_metabolism; XBM: Xenobiotics_biodegradation_and_metabolism; The letters represent the group with significantly higher abundance.

**Appendix D Comparison of Alpha diversity indices of gut microbiota from different groups.**

| Group | observed_otus | pielou_e | shannon |
| --- | --- | --- | --- |
| SCM | 427.5±111.38 | 0.89±0.02 | 7.71±0.53 |
| WCM | 775.75±89.30 | 0.88±0.01 | 8.40±0.24 |
| SCF | 320.67±10.37 | 0.86±0.03 | 7.20±0.23 |
| WCF | 763.00±49.07 | 0.88±0.002 | 8.45±0.10 |
| SWF | 525.60±55.64 | 0.89±0.02 | 8.05±0.24 |
| WWF | 908.15±116.82 | 0.90±0.01 | 8.84±0.27 |
| SWM | 536.50±36.88 | 0.88±0.01 | 8.02±0.21 |
| WWM | 867.88±94.03 | 0.90±0.01 | 8.76±0.21 |
